# Terahertz Wave Absorption Property of all Mixed Organic–Inorganic Hybrid Perovskite Thin Film MA(Sn, Pb)(Br, I)_3_ Fabricated by Sequential Vacuum Evaporation Method

**DOI:** 10.3389/fchem.2021.753141

**Published:** 2021-09-16

**Authors:** Inhee Maeng, Hiroshi Tanaka, Valynn Katrine Mag-usara, Makoto Nakajima, Masakazu Nakamura, Min-Cherl Jung

**Affiliations:** ^1^ YUHS-KRIBB, Medical Convergence Research Institute, College of Medicine, Yonsei University, Seoul, South Korea; ^2^ Division of Materials Science, Nara Institute of Science and Technology, Ikoma, Japan; ^3^ Institute of Laser Engineering, Osaka University, Suita, Japan; ^4^ Division of Materials Science, Faculty of Pure and Applied Sciences, University of Tsukuba, Ibaraki, Japan

**Keywords:** sequential vacuum evaporation, atomic structure, chemical state, THz-wave absorption, MA(Sn, Pb)(Br I)_3_

## Abstract

All mixed hybrid perovskite (MA(Sn, Pb)(Br,I)_3_) thin film was fabricated by sequential vacuum evaporation method. To optimize the first layer with PbBr_2_ and SnI_2_, we performed different annealing treatments. Further, MA(Sn, Pb)(Br, I)_3_ thin film was synthesized on the optimized first layer by evaporating MAI and post-annealing. The formed hybrid perovskite thin film exhibited absorptions at 1.0 and 1.7 THz with small absorbance (<10%). Moreover, no chemical and structural defect-incorporated absorption was found. In this study, the possibility of changing terahertz absorption frequency through the mixture of metal cations (Sn^+^ and Pb^+^) and halogen anions (Br^−^ and I^−^) was verified.

## Introduction

Recently, organic–inorganic hybrid perovskite (OHP) materials, because of their universal properties such as controllable bandgap, weak exciton binding energy, and high-carrier mobility, are attracting great attention for solar-cell and light-emitting diode applications (D’Innocenzo et al., 2014; Frost et al., 2014; Frost and Walsh, 2016; Pedesseau, 2016; NR Venkatesan, 2018; Yi et al., 2019). Many researchers are focusing on their fundamental properties to explore possibilities of new applications such as laser, memory device, and terahertz (THz) detector (ER Dohner, 2014; Kepenekian et al., 2015; Saba et al., 2016; Srimath Kandada and Petrozza, 2016; Lee et al., 2018; Sarritzu et al., 2018; Straus and Kagan, 2018; Grancini and Nazeeruddin, 2019; Obraztsov et al., 2021). In fact, the versatility of OHP contains very high possibility in novel device applications. One of new possibilities using OHP is THz-based application because OHP is composed of perovskite structure between organic and inorganic portions. In principle, the OHP structure is supposed to possess both molecular vibrations from the organic portion and lattice vibrations from the inorganic portion (Park et al., 2016).

To realize a THz-based application using OHP materials, two phonon modes such as molecule cation and metal cation–halogen anion vibrations in the THz range from 0.5 to 3 THz are primarily investigated (La-O-Vorakiat et al., 2016; Zhao et al., 2017; Cinquanta et al., 2019; Lee et al., 2019; Maeng et al., 2020b, 2020a). Further, the molecular motions (rotation and translation) of the CH_3_NH_3_^+^ (MA^+^) ion, which were confirmed by powder neutron diffraction, were found to be ordered, whereas the 2D and 3D disorder was dependent on each structure being controlled by temperature (Weller et al., 2015). However, there is no report on the experimental observation of the molecular vibration and rotation in CH_3_NH_3_PbI_3_ (MAPbI_3_) and *α*-HC(NH_2_)_2_PbI_3_ (*α*-FAPbI_3_) thin films (Lee et al., 2019; Maeng et al., 2019). Possibly, the contribution of molecular motions is considerably weak in the THz energy range from 0.5 to 3 THz. In MAPbI_3_ thin films fabricated by a conventional solution-based method, two vibrational modes at 1 and 2 THz, originated from the buckling of the Pb–I–Pb angles and the Pb–I bond vibration, respectively, were observed (La-O-Vorakiat et al., 2016). Additionally, a high THz-wave absorption at 1.58 THz was observed in MAPbI_3_ thin film fabricated by sequential vacuum evaporation (SVE) method, which incorporated CH_3_NH_2_ molecular defects in the synthesized thin film (Maeng et al., 2019; 2020b). Our recent reports indicate that a vacuum-processed method such as SVE creates a greater number of variable atomic and chemical states than a solution-processed method because vacuum-processed methods produce smaller grains, a higher density of grain boundaries and defects (Jung et al., 2018; Lee et al., 2019; Maeng et al., 2020a, 2020b). Generally, the thin films formed by solution-processed methods such as 2-step and antisolvent methods are shown with two significant features such as large grain size (>500 nm) and low density of grain boundary that are induced with high carrier mobility in a solar-cell application (Ono and Qi, 2016, 2018). In the case of the vacuum-processed methods such as co-evaporation and SVE, it shows a relatively small grain size (<200∼300 nm) and a high density of grain boundary. The annealing process is very important to improve the quality of thin films fabricated by vacuum-processed methods. However, it causes to deplete elements on the surface and makes an unstable stoichiometry. (Ono and Qi, 2016). Also, the grain size does not improve significantly (Lee et al., 2019; Maeng et al., 2020a, 2020b).

To confirm the origins of various THz absorptions in OHP thin film, different composition elements for metal cation and halogen anion such as Sn and Br need to be investigated. In addition, a study on OHP thin films with mixtures of metal cations as well as halogen anions will be significantly interesting because of a modulation possibility of absorption frequency which can be an essential function for THz-detector or -modulator.

In this study, we fabricated and characterized an all mixed hybrid perovskite, MA(Sn, Pb)(Br, I)_3_. First, we optimized the first layer with lead (II) bromide (PbBr_2_) and tin (II) iodide (SnI_2_) using SVE method. Next, MA(Sn, Pb)(Br, I)_3_ thin film was formed by evaporating MAI in the same method. In the THz time domain spectroscopy (THz-TDS), the formed hybrid perovskite thin film exhibited 1.0 and 1.7 THz absorptions, and there was no chemical and structural defect-incorporated absorption.

## Material and Methods

**FIGURE 1:**
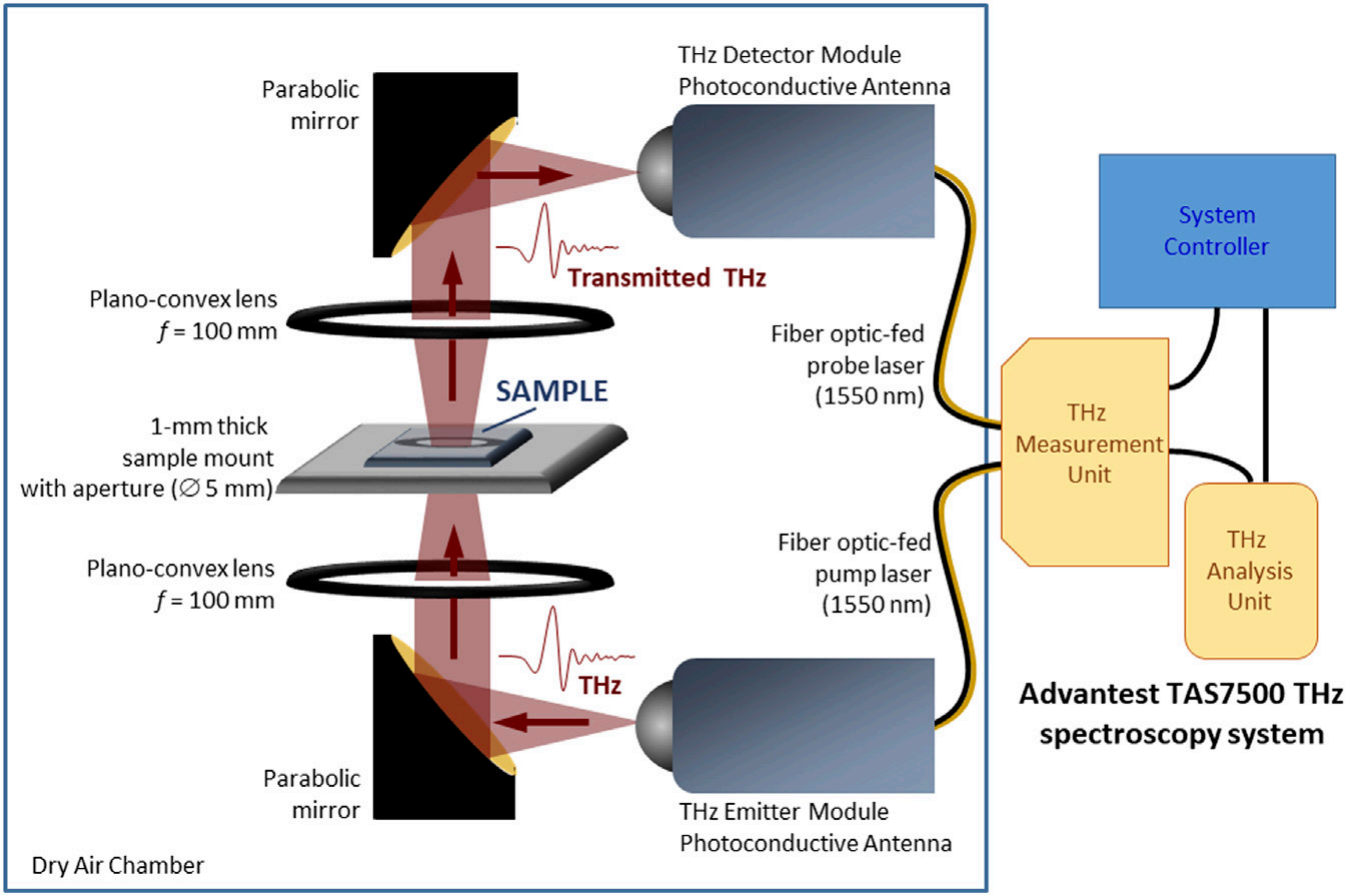
The transmission terahertz time-domain spectroscopy (THz-TDS) system used for investigating the transmittance of the hybrid perovskite samples (This schematic is not drawn to scale.)

OHP thin films were fabricated by SVE in a customized vacuum chamber. A polyethylene terephthalate (PET) flexible substrate (AHCF-100, thickness = 225 μm, AIDEN) was cleaned by sonication in acetone for 10 min, rinsed in heated acetone for 1 min, and then treated in a UV-ozone chamber for 30 min before finally loading it into a vacuum chamber. To fabricate all mixed organic–inorganic hybrid perovskite thin film using SVE, first, we optimized the first mixed layers with SnI_2_ and PbBr_2_. The SVE chamber had two heaters for separate evaporations of SnI_2_ and PbBr_2_. The base pressure was 8.0 × 10^–3^ Pa. First, SnI_2_ (Sigma-Aldrich, 99.99% purity) was evaporated onto the substrates at room temperature with a fixed deposition rate and thickness of 10 Å/s and 50 nm, respectively, (Jung et al., 2018). Next, we evaporated PbBr_2_ (Sigma-Aldrich, > 99.8% purity) onto the substrates with same deposition rate and thickness (Jung et al., 2018). Finally, we performed separate post-annealing at 110°C for 10, 20, and 45 min, respectively, of three fabricated films in an N_2_ glovebox to find an optimized mixed thin film (Figure 1).

From this first optimization, we selected the sample post-annealed for 10 min. The mixed layer with SnI_2_/PbBr_2_ was further deposited with CH_3_NH_3_I (Methylammonium iodide, MAI, Sigma-Aldrich, 98% purity) with a fixed deposition rate and thickness of 2 Å/s and 300 nm, respectively, (Jung et al., 2018). Again, we performed separate post-annealing at 110°C for 10, 20, and 45 min, respectively, in an N_2_ glovebox to form final organic–inorganic hybrid perovskite structures.

To protect the formed thin films against air and water, finally, spin coating was applied at 4 krpm for 1 min to coat all thin films with a PTAA (Sigma-Aldrich) solution containing 5 mg PTAA and 4 ml chlorobenzene. The PTAA solution was sonicated for 1 h (Lee et al., 2019; Maeng et al., 2020a).

To characterize the formed thin films, we performed scanning electron microscopy (SEM), X-ray diffraction (XRD), and X-ray photoelectron spectroscopy (XPS). SEM characterization was conducted at an acceleration voltage of 5 keV and an emission current of 10 μA using HITACHI SU9000. The XRD instrument was RINT-TTRIII/NM with a Cu *K*_*α*_ source constructed by Rigaku. All XPS measurements were performed using Versa ProbeII with a monochromated Al*K*_α_ (ULVAC-PHI) to obtain Pb 4*f*, I 4*d*, Sn 3*d*, Br 3*d* core-levels, and valence spectra. Binding energies were calibrated with respect to the Au 4*f*_7/2_ core-level (84.0 eV) (Wagner et al., 1995).

Transmission type THz-TDS was utilized to characterize the samples and elucidate their THz-wave absorption properties. (Nakajima et al., 2008; Nakajima et al., 2009; Nakajima et al., 2016; Jung et al., 2018; Ohkoshi et al., 2020; Agulto et al., 2021; Fitzky et al., 2021). Transmittances of the samples were obtained by non-destructive testing and analysis using an Advantest TAS7500 THz spectroscopy system, which allowed the direct measurement of time-domain THz waveforms and the corresponding power spectra at room temperature (22 ± 1°C) and dry air (relative humidity <1.5%) conditions. The system, which had a spectral resolution of 7.6 GHz, was equipped with a photoconductive antenna (PCA) for THz emission, a sample holder, and another PCA for the detection of transmitted THz radiation through the sample. For all THz transmission measurements, each sample was mounted on a sample holder with 5-mm-diameter aperture at a fixed optimum distance between the two PCAs, and measurements with only the substrate as well as without any sample on the sample holder were also performed for reference. The transmittance was subsequently acquired by comparing the transmission spectra of each sample with the corresponding reference spectra. The schematic of the THz-TDS system is shown in Figure 1.

## Results and Discussion

**FIGURE 2:**
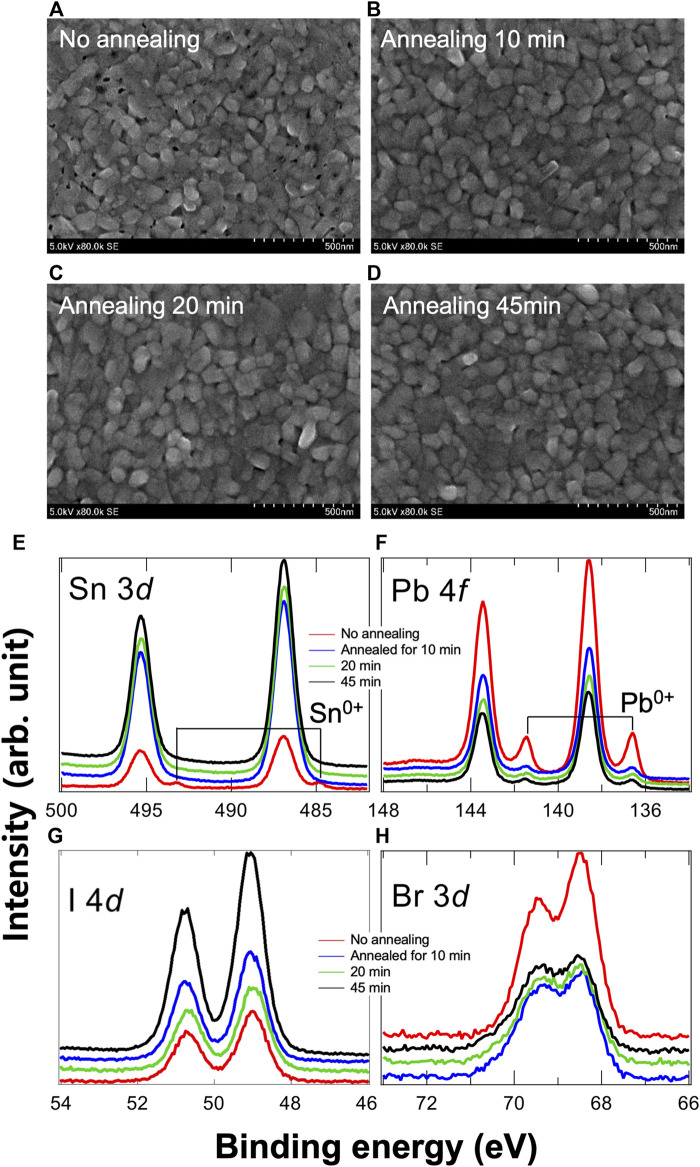
Surface morphologies of PbBr_2_/SnI_2_ after **(A)** no-annealing, **(B)** 10-min annealing, **(C)** 20-min annealing, and **(D)** 45-min annealing treatments. The grain sizes do not show any significant change. Additionally, the vacant area disappears after annealing. Chemical states of **(E)** Sb 3*d*, **(F)** Pb 4*f*, **(G)** I 4*d*, and **(H)** Br 3*d* core-levels. Because of the annealing process, we confirm that a physical mixture of PbBr_2_/SnI_2_ is formed because there is no new chemical state, and changes in the peak intensities only are observed.

To find an optimized mixed thin film with PbBr_2_/SnI_2_, we performed the post-annealing with different annealing times. Drastic changes in the surface morphologies and chemical states after the annealing treatments are shown. (Figure 2). From the SEM results, it can be observed that the grain sizes, which are in the range of 80–120 nm, do not change significantly for different post-annealing times. However, the vacant areas disappeared with increased post-annealing time. (Figures 2A–D). Interestingly, all XPS intensities change dramatically for the samples annealed for 10 min and more. The intensities corresponding to Sn 3 and I 4*d* core-levels increase, (Figures 2E,G), whereas the intensities corresponding to Pb 4*f* and Br 4*d* core-levels decrease, (Figures 2F,H), indicating that the bottom SnI_2_ and upper PbBr_2_ are out- and in-diffused to the surface and bulk, respectively. As the annealing time increases, this trend becomes more prominent. Notably, no new chemical state is observed during annealing. Based on these observations, we confirmed the following:

1) Initially formed thin film structure had physical separation between SnI_2_ and PbBr_2_.
2) The post-annealing process induced a physically mixed layer of SnI_2_ and PbBr_2_.

Additionally, the chemical states of Sn^0+^ and Pb^0+^ were observed for the no-annealing sample. However, they soon disappeared as the post-annealing process was initiated. Although there was still a small trace of Pb^0+^ after annealing, the annealing process almost induced a depletion of non-stoichiometry elements in the thin film. For the next deposition step, the optimized thin film is required with a rough stacking structure (Jung et al., 2018). We selected the 10-min annealing sample because if the thin film was dense, the second element, MAI, would not be able to penetrate the bulk (Jung et al., 2018).

**FIGURE 3:**
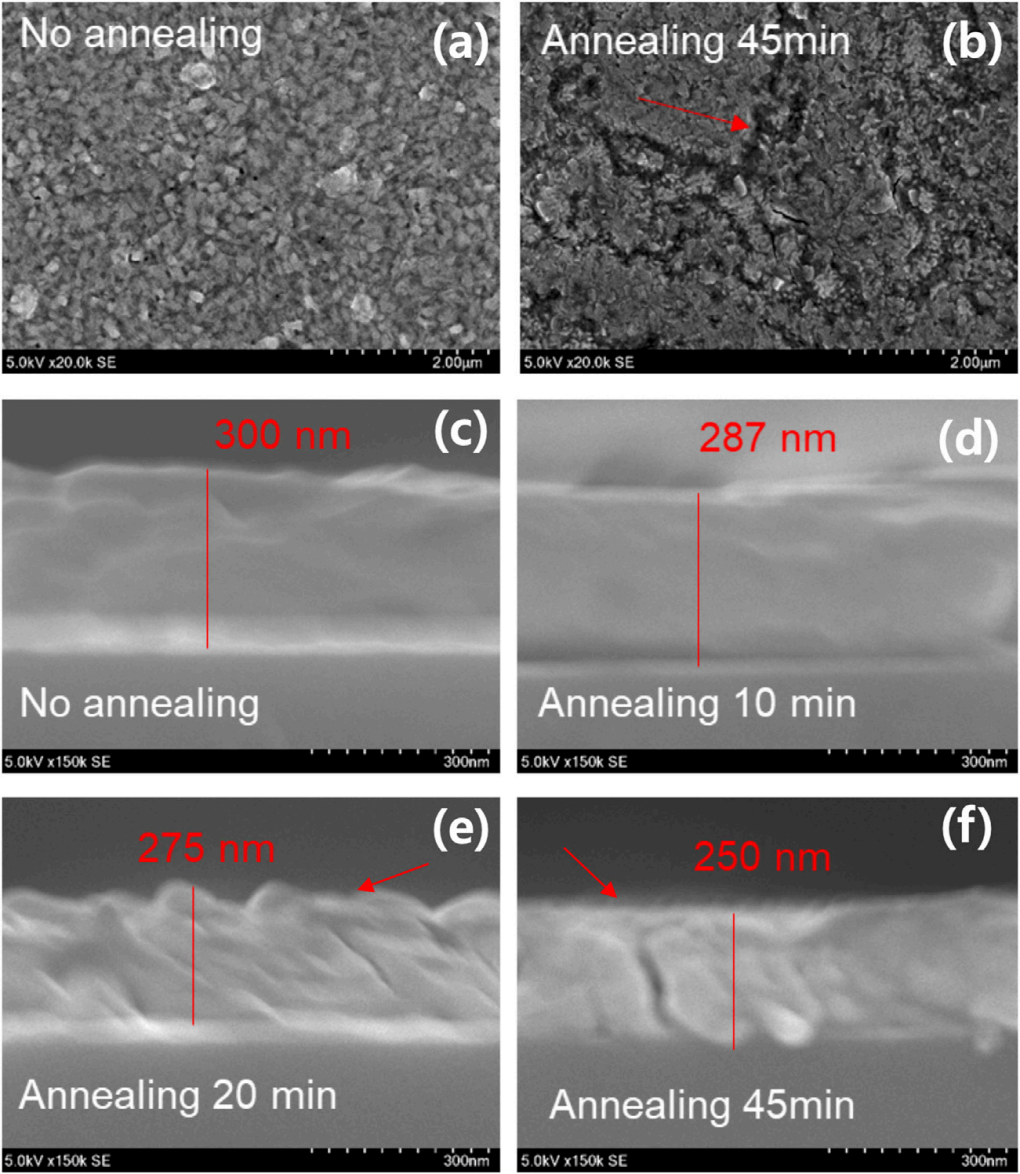
Surface morphologies after the deposition of MAI of **(A)** no-annealing and **(B)** 45-min annealing samples. The surface is dramatically changed with the appearance of a valley. **(C–F)** Additionally, the thickness decreases from 300 to 250 nm with increase in the annealed time. The deep void can be observed in both the 20- and 45-min annealing samples.

To obtain all mixed OHP thin film, we performed the post-annealing with different annealing times such as 10, 20, and 45 min after evaporating MAI. We observe different surface morphologies for the no- and 45-min annealing samples. (Figures 3A,B). For the 45-min annealing sample, we notice a profound crack on the surface (indicated by the red arrow). (Figure 3B). Interestingly, the film thickness, along the cross-sectional view of the thin film, decreases from 300 to 250 nm as the annealing time increases. (Figures 3C–F). Moreover, we confirm that the observed crack in the 45-min annealing sample reaches from surface to bulk (red arrow). This crack started to appear in the 20-min annealing sample. Interestingly, we did not find any cracked structure in the 10-min annealing sample.

**FIGURE 4:**
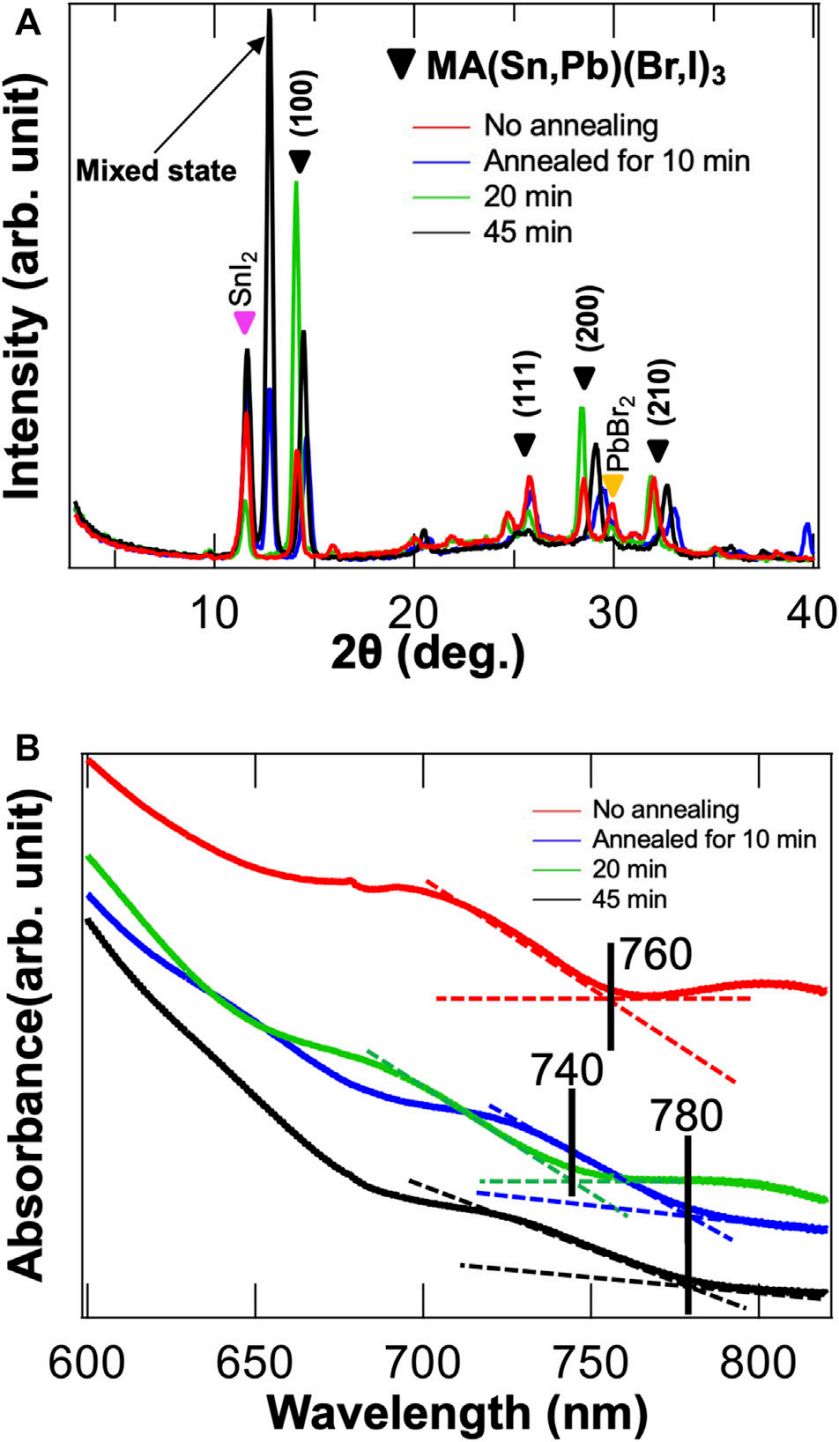
**(A)** XRD results. The SnI_2_ and PbBr_2_ are still present. In the 10-min annealing sample, the perovskite structure is observed. The mixed states are remarkably prominent in the 45-min annealing sample. **(B)** Optical band gap is approximately measured with 1.65 eV in UV-Vis spectrometer.

To confirm the atomic structure, we performed XRD and UV-Vis measurements. (Figure 4). Interestingly, SnI_2_ and PbBr_2_ components are still observed in the XRD results. The mixed state, which was incorporated with the CH_3_NH_2_ molecular defect, increases dramatically for an annealing period of 20 min and more (Jung et al., 2018; Maeng et al., 2020b, 2020b). From these results, we can confirm that owing to the presence of cracks and remaining constituent elements, the formation of all mixed perovskite structure in a thin film is difficult using SVE. However, the 10 and 20-min annealing samples exhibited relatively small intensities for SnI_2_, PbBr_2_, and mixed state. Unfortunately, it is very difficult to determine an exact stoichiometry of our formed hybrid perovskite thin film from XRD results. In the UV-Vis measurements, the optical absorptions of the no-annealing, 10-min, 20-min, and 45-min annealing samples are 760, 740, 780, and 780 nm, respectively, (Figure 4B). We did not observe any change in the optical absorption after the 20-min annealing treatment.

**FIGURE 5:**
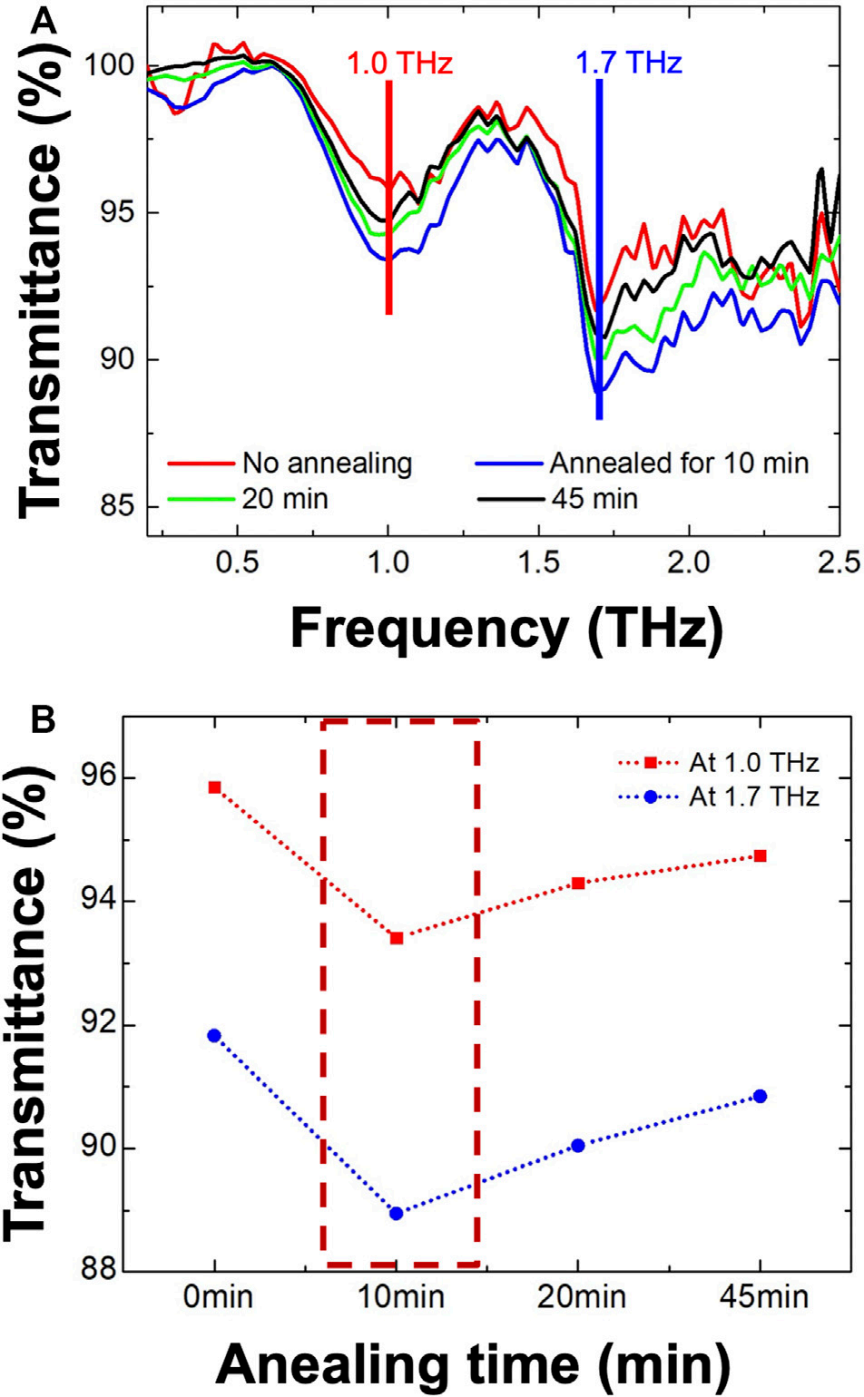
**(A)** Major THz-wave absorptions are measured at 1.0 and 1.7 THz for each annealed sample. Interestingly, **(B)** the 10-min annealing sample shows the maximum transmittance with 93.5 and 89% at 1.0 and 1.7 THz, respectively.

To explore the THz-wave absorption property, we performed THz-TDS experiment. Although the absorptance does not appear to be strong, we observe two prominent absorptions at 1.0 and 1.7 THz for all samples. (Figure 5A). After annealing for 10 min, the absorption property improves slightly. (Figure 4B). However, the transmittance increases as the annealing time is increased further. (Figure 5B). Consistently with the XRD results, it shows a degradation of perovskite structure after the 10-min annealing process. According to our previous studies, the 1.0 and 1.7 THz absorptions are assumed to originate from buckling and translation vibrations, respectively, (Maeng et al., 2019; 2020a). However, we did not observe any additional absorption such as defect-incorporated structure or Br self-vibration. (Maeng et al., 2019; 2020a). To compare with MAPbI_3_ and MAPbBr_3_, we have summarized various parameters related to THz-wave absorption in Table 1.

**TABLE 1:** Comparison of THz-wave absorption properties of each perovskite thin film.

Sample	Peak 1 (buckling vibration)	Peak 2 (translation vibration)	Peak 3	Lattice constant (Å)	Crystal structure (RT)
MA(Sn,Pb) (Br,I)_3_	1.0 THz	1.7 THz	No peak	6.20	Cubic
MAPbI_**3**_ Maeng et al. (2019)	0.95 THz	1.87 THz	1.58 THz (Defect-incorporated)	6.27	Tetra
MAPbBr_3_ Maeng et al. (2020a)	0.85 THz	1.38 THz	2.00 THz (Br self-vibration)	5.95	Cubic
MASnI_3_ Li and Rinke. (2016)	There is no significant THz-wave absorption			6.23	Cubic

Interestingly, the lattice constant of the formed MA(Sn, Pb)(Br,I)_3_ is 6.20 Å, which is an intermediate value between the lattice constant values of MAPbI_3_ (6.27 Å) and MAPbBr_3_ (5.95 Å). Furthermore, we have confirmed that the THz-wave absorptions of MA(Sn, Pb)(Br,I)_3_ are different in comparison with those of MAPbI_3_ and MAPbBr_3_. (Table 1). Unfortunately, we did not observe any significant THz-wave absorption in MASnI_3_, indicating a lack of clarity regarding the effect of Sn. In short, the origin of this increased absorption frequency is not easy to understand. However, we demonstrated that the fabricated mixed hybrid perovskite changes the frequencies of THz-wave absorptions.

## Conclusion

We fabricated MA(Sn, Pb)(Br, I)_3_ by SVE in this study. Although the formed thin films were subjected to different annealing treatments, further optimizations are required in the thin film fabrication process. However, we verified the possibility of THz-wave absorption changes due to the mixture of metal cations (Sn^+^ and Pb^+^) and halogen anions (Br^−^ and I^−^). To be a candidate material for THz-based application such as sensing and modulating devices, we believe this confirmation is a first step for the frequency modulating by the element mixtures.

## Data Availability

The raw data supporting the conclusions of this article will be made available by the authors, without undue reservation.
